# Improving the demand for birth registration: a discrete choice experiment in Ethiopia

**DOI:** 10.1136/bmjgh-2019-002209

**Published:** 2020-05-21

**Authors:** Mahari Yihdego, Ayanaw Amogne, Selamawit Desta, Yoonjoung Choi, Solomon Shiferaw, Assefa Seme, Li Liu, Stéphane Helleringer

**Affiliations:** 1 PMA Ethiopia Project, Addis Ababa University, Addis Ababa, Addis Ababa, Ethiopia; 2 School of Public Health, Johns Hopkins University, Baltimore, Maryland, USA; 3 iSquared, Information x Insight, Severna Park, MD, USA; 4 Department of Reproductive Health and Health Service Management, Addis Ababa University, Addis Ababa, Ethiopia

**Keywords:** child health, cross-sectional survey, other study design

## Abstract

**Introduction:**

Birth registration remains limited in most low and middle-income countries. We investigated which characteristics of birth registration facilities might determine caregivers’ decisions to register children in Ethiopia.

**Methods:**

We conducted a discrete choice experiment in randomly selected households in Addis Ababa and the Southern Nations, Nationalities, and People’s Region. We interviewed caregivers of children 0–5 years old. We asked participants to make eight choices between pairs of hypothetical registration facilities. These facilities were characterised by six attributes selected through a literature review and consultations with local stakeholders. Levels of these attributes were assigned at random using a fractional design. We analysed the choice data using mixed logit models that account for heterogeneity in preferences across respondents. We calculated respondents’ willingness to pay to access registration facilities with specific attributes. We analysed all data separately by place of residence (urban vs rural).

**Results:**

Seven hundred and five respondents made 5614 choices. They exhibited preferences for registration facilities that charged lower fees for birth certificates, that required shorter waiting time to complete procedures and that were located closer to their residence. Respondents preferred registration facilities that were open on weekends, and where they could complete procedures in a single visit. In urban areas, respondents also favoured registration facilities that remained open for extended hours on weekdays, and where the presence of only one of the parents was required for registration. There was significant heterogeneity between respondents in the utility derived from several attributes of registration facilities. Willingness to pay for access to registration facilities with particular attributes was larger in urban than rural areas.

**Conclusion:**

In these regions of Ethiopia, changes to the operating schedule of registration facilities and to application procedures might help improve registration rates. Discrete choice experiments can help orient initiatives aimed at improving birth registration.

**Key questions**

**What is already known?**

- Birth registration is incomplete in most low and lower-middle income countries.
- This limits access to rights, protections and services for children.
- It also prevents the establishment of reliable vital statistics about fertility and mortality.

**What are the new findings?**

- In two regions of Ethiopia, there were important barriers to birth registration related to costs, distances and wait times.
- Caregivers of young children also expressed strong preferences for registration facilities that had convenient opening schedules (eg, evenings and weekends), and delivered birth certificates in a single visit.
- In urban areas, only requiring one of the parents to be present at the time of registration might also help improve registration rates.

**What do the new findings imply?**

- Interventions that modify the opening schedule of registration facilities, as well as registration procedures, might complement current initiatives to improve birth registration.
- Discrete choice experiments have the potential to help inform the development of birth registration systems in low and middle-income countries.

## Introduction

Birth registration is the process of recording a child’s birth in governmental registers or databases. It is necessary to establish a birth certificate, which gives each child a number of rights and protections.^1 2^ For example, it helps establish filiation and inheritance rights. Ownership of a birth certificate is associated with fewer school dropouts, reduces exposure to child trafficking, labour or early marriage and often improves access to healthcare services.^3–5^ Birth registration is also a key component of the production of annual estimates of fertility and mortality rates. These vital statistics are essential in planning and evaluating social services such as healthcare or education.^3 6 7^

The coverage of birth registration varies greatly throughout the world.^6^ In high-income countries, birth registration is timely and (nearly) universal. In poorer countries, many births are never registered.^3^ Others are registered only several years after the birth, for example, when a birth certificate is needed to enrol in school. Within countries, the most disadvantaged social groups have lower registration rates than more affluent groups.^8–10^

Reaching universal birth registration in low income and lower-middle income countries (LLMIC) has recently become a key objective of LMICgovernments and various global actors.^11 12^ The birth registration rate is one of the indicators used to track progress towards the 16th and 17th Sustainable Development Goals, that is, the promotion of more inclusive societies and the strengthening of systems contributing to sustainable development. Major global initiatives have been launched to strengthen civil registration and vital statistics (CRVS) systems.^13 14^ They focus on promoting legislative changes required to expand birth registration, developing new tools to facilitate the production of vital statistics and/or strengthening the administrative systems that implement birth registration.

Improving the coverage of birth registration in LMICs also requires stimulating the demand for, and removing barriers to, birth registration among local populations, particularly in settings where significant numbers of births occur at home. This is so because CRVS systems are predominantly ‘passive’: the caregiver(s) of a child must contact a CRVS agent to report the occurrence of the birth and complete the required paperwork.

We investigated the preferences of caregivers for the registration of births in two regions of Ethiopia, a country with some of the lowest birth registration rates worldwide.^15^ We used a discrete choice experiment (DCE), that is, a survey methodology in which respondents repeatedly choose between hypothetical versions of a service characterised by a small number of randomly selected attributes.^16^ Statistical analysis of DCE data allows assessing the relative importance of each of these attributes in influencing decisions to obtain a particular service. DCEs are widely used in marketing and management,^17 18^ and have recently helped guide health systems strengthening in LMICs.^19–23^ This methodology has however not been used to inform the development of CRVS systems.

## Methods

### Study context

**Figure 1:**
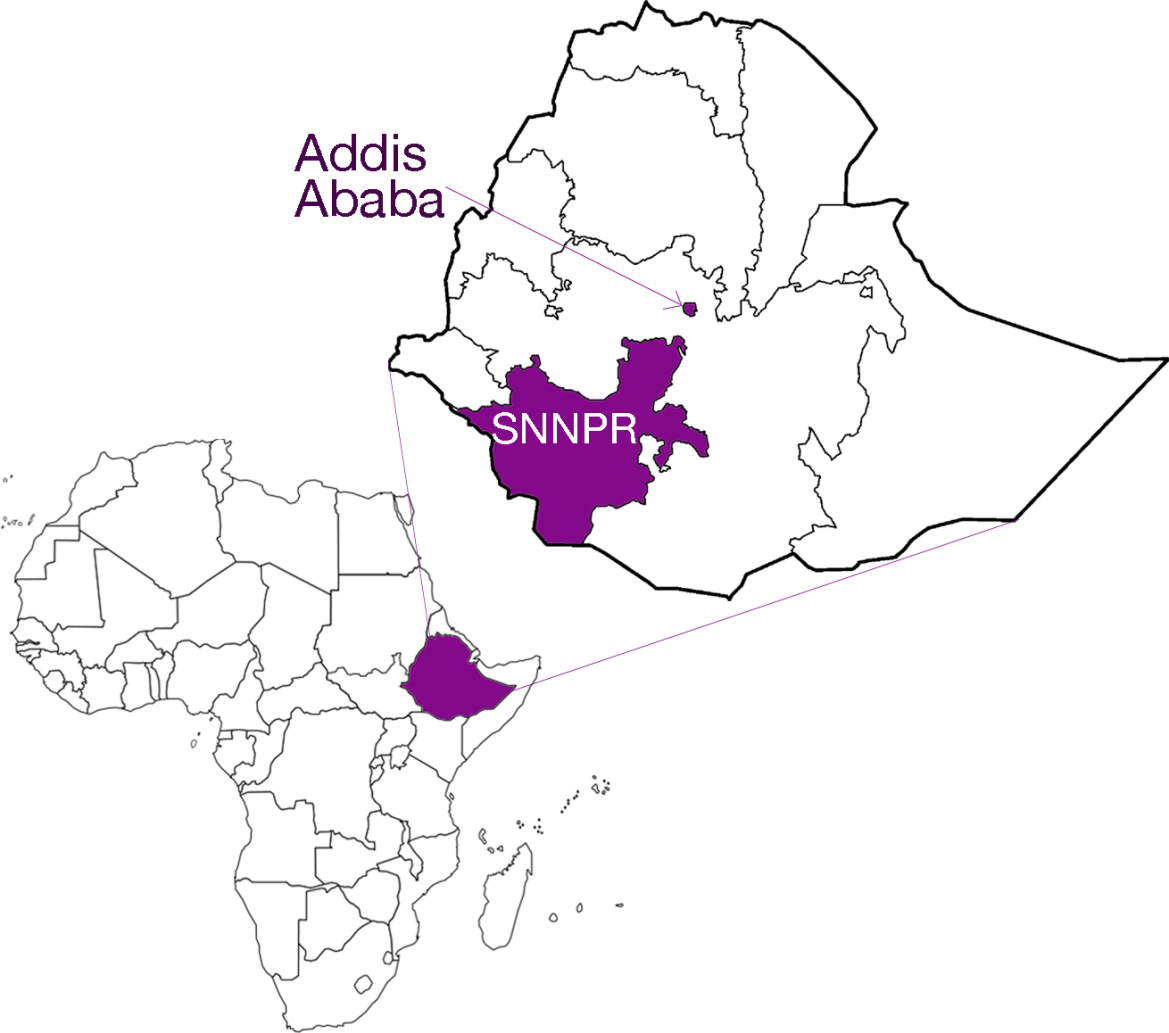
Map of the regions included in the birth registration study. SNNPR, Southern Nations, Nationalities, and People’s Region.

This study is part of Performance Monitoring for Action (PMA), a multicountry project that collects survey data on key health indicators.^24^ We worked in Ethiopia, a country of more than 105 million inhabitants in East Africa (figure 1). Ethiopia is a low-income country: in 2018, its gross domestic product was US$772.3 per capita, according to World Bank estimates. Ethiopia has one of the lowest birth registration rates in Eastern and Southern Africa, with approximately 3% of children under age 5 registered in.^15^ By comparison, a third of under-5 children are registered in nearby Uganda,^25^ and more than two-thirds are registered in neighbouring Kenya.^26^ PMA has conducted nationally representative surveys in Ethiopia since 2013, with a focus on family planning, maternal/newborn health and water/sanitation.^27 28^

Several months after the sixth round of PMA data collection (‘R6 survey’ thereafter), we conducted a follow-up study of birth registration (‘Birth registration study’ thereafter) in two regions: Addis Ababa and Southern Nations, Nationalities, and People’s Region (SNNPR; figure 1). Addis Ababa is an urban region with 3.2 million inhabitants, according to projections based on data from the 2007 census. SNNPR is one of the most populous regions, with 17.9 million inhabitants. It borders Kenya and South Sudan to the south and west, respectively. It is predominantly rural, but it also includes several large cities of >100 000 inhabitants. According to the most recent Demographic and Health Survey (2016), 24% of children under-5 had their birth registered in Addis Ababa, the highest registration rate in the country. In comparison, 3% of children under-5 in SNNPR had their birth registered, on par with the national average.^15^

In Ethiopia, the Vital Events Registration and National Identification proclamation of 2012 (revised in 2017) is the law that regulates the administrative process of birth registration. The federal Vital Events Registration Agency (VERA) was established in 2014 to oversee this process. The implementation of civil registration (including births) under the new law began nationwide in 2016.^29^

Health facilities, as well as health extension workers who routinely visit households, are expected to produce notification forms for births. These forms contain information about the child (name, date of birth). They do not replace the forms and certificates that must be obtained from the registration offices located in each kebele, that is, the lowest administrative unit in the country.

There are more than 18 000 kebeles in Ethiopia, the large majority of which now offer birth registration services.^30^ Kebele offices are often accessible for most of the population they serve, particularly in urban areas. In some of the rural and mountainous parts of the SNNPR, however, households may be located several hours away from their kebele office.^31^ The level of staffing and equipment of kebele offices also varies between urban and rural areas: in Addis Ababa and other urban areas of the country, civil registration officers in a growing proportion of kebele offices use computers to register births or issue certificates, whereas paper forms remain the norm in virtually all rural areas.

Birth registration is free in Ethiopia, but families might be charged a fee to obtain their child’s birth certificate, with the amount of the fee set by each administrative region. Birth registration is mandatory and should be accomplished within 90 days of birth. After that delay, penalties might be incurred but are rarely enforced in practice. The parents of a child must both be present at the registration office, and they must show their identification card to register the birth of their child. If one or both parents cannot be present to register a birth, additional procedures (eg, affidavits, sworn statements) are required to allow the available parent, or a guardian, to carry out birth registration.

### Study participants

For the birth registration study that included the DCE, we selected a subset of households that had participated in the R6 survey. The R6 survey was conducted in June and July 2018. It used a two-stage cluster design, with urban-rural and administrative regions as strata. In the first stage, 44 EAs were selected in the SNNPR, and 22 EAs in Addis Ababa. Urban EAs were oversampled in the SNNPR. In the second stage, 35 households were selected at random within each EA. In total, 1617 households in the SNNPR, and 761 households in Addis Ababa, participated in the R6 survey.

Households were eligible for the birth registration study if they had a child aged 0–5 years among their members. We revisited selected households between December 2018 and March 2019. At that time, we confirmed the presence of children 0–5 years old using a household roster. We determined which household member was the primary caregiver of each listed child, that is, the parent or legal guardian. We selected study participants among adult caregivers. If there was only one primary caregiver in the household, he/she was automatically selected. If there were multiple primary caregivers in a household, we selected one at random. Households in three rural EAs in SNNPR could not be included due to security reasons.

### Study questionnaire

The birth registration study consisted of a face-to-face interview with selected caregivers. In addition to the DCE module, the questionnaire ascertained a caregiver’s demographic characteristics, his/her knowledge of birth registration, the registration status of the children he/she cares for and exposure to messages stressing the need to register births.

### DCE design

The DCE was designed to estimate the relative value that caregivers assign to attributes of birth registration facilities in considering whether and where to register their child(ren). We first conducted a review of the literature on the barriers to birth registration in LMICs. The protocol of this review is provided in online supplementary file 1. Based on review results, we identified several barriers that are characteristics of the registration process or the facilities that carry out this process. We determined which of these barriers were relevant to the Ethiopian context through a review of legislative documents (ie, the 2012 and 2017 proclamations), and consultation with VERA officials.

10.1136/bmjgh-2019-002209.supp1 Supplementary data

This process yielded six DCE attributes (table 1): (1) the cost of obtaining a birth certificate, (2) the time to wait for service at the registration facility, (3) the number of visits required to register a birth and obtain the birth certificate, (4) the opening schedule of the registration facility, (5) the distance to the registration facility from the caregiver’s residence, and (6) whether the presence of one or both parents is required to complete the registration. For each attribute, we selected two to three levels that were either representative of the situation of birth registration in Addis Ababa and SNNPR or constituted desirable alternatives. We piloted the DCE design with stakeholders, potential data collectors and participants. Based on feedback, we refined the definition and levels of each attribute, and we developed training instructions.

**Table 1:** Attributes and levels of registration facilities used in the discrete choice experiment, Addis Ababa and SNNPR of Ethiopia 2018/2019

Attributes	Levels
Cost of birth certificate	Free (0 birr)
	100 birr
	250 birr
Time to wait for service at registration facility	1 hour
	3 hours
	5 hours
Distance to registration facility	30 min walk
	2-hour walk
	4-hour walk
Number of visits required to register birth	Single visit
	Multiple visits
Opening hours of the registration facility	Regular hours (weekdays, 08:30–17:00)
	Extended hours (weekdays, 08:30–19:00)
	Weekend hours (weekdays, 08:30–17:00+Saturdays, 08:30–12:00)
Application procedures	Only one parent present Both parents present

SNNPR, Southern Nations, Nationalities, and People’s Region.

There were 324 potential combinations of the attributes and levels described in table 1. Respondents could not evaluate each of these combinations. Instead, we asked them to make eight choices between two randomly selected hypothetical registration facilities. The alternatives in each of the eight choice sets were formed using the DCREATE module in Stata, which creates efficient fractional designs.^32^ This approach allows assessing preferences for each level of the attributes. Our DCE was unlabelled,^33^ with alternatives presented to respondents under the headings of ‘facility A’ and ‘facility B’. In each of the choice sets, we also gave respondents the option to select neither facility. This ‘opt-out’ option helps increase the external validity of DCE data because respondents are not forced to choose between two (possibly unrealistic) alternatives.^34 35^

In addition, the DCE included two practice choice sets, during which interviewers demonstrated DCE procedures, verified respondents’ understanding of DCE procedures and addressed questions. We also added a choice set that contained a ‘dominant’ alternative, that is, one of the two hypothetical facilities was preferable to the other facility on all attributes.^36^ This choice set was inserted to evaluate the respondents’ comprehension of DCE procedures. Based on feedback obtained during the pilot, we randomly placed it in the sequence of choice sets to avoid instances where interviewers would select the dominant choice themselves to save time, instead of asking respondents to make the choice. Finally, we randomly varied between respondents the order in which attributes were listed in each choice set. This allowed assessing whether respondents made decisions based on the value of the attributes that were listed first. Prior studies have used similar checks to establish the reliability of DCE data.^19 20 37^

### Sample size

We determined the sample size of the birth registration study to estimate indicators of CRVS coverage with a desired level of precision. For analyses of DCE data, given large differences between urban and rural areas in (A) characteristics of respondents, and (B) accessibility and equipment of kebele offices, we sought to elicit caregivers’ preferences separately by place of residence. According to formulas of the statistical power of DCEs,^38^ sample sizes from the birth registration study in these sampling strata were sufficient to estimate the main effects of each facility attribute on respondents’ choices.

### Data collection

We administered the questionnaire with Open Data Kit, a data collection platform frequently used in LMICs.^39^ We translated study instruments into Amharic. We trained data collectors for 5 days on study procedures. Interviewers first read a script explaining DCE procedures to respondents. Then, they stated the levels of the attributes of each hypothetical facility included in a choice set. They repeated these attributes if necessary, and encouraged respondents to take their time in making each DCE choice.

We built automated quality checks into the DCE module. We flagged instances where a respondent opted out (ie, selecting neither facility), or selected the same facility (eg, facility A), in each choice set. Field supervisors were alerted to those occurrences and were asked to provide feedback to interviewers. In some cases, they revisited respondents for verification and corrections, if needed.

### Statistical analysis

We tabulated the characteristics of caregivers, by place of residence (urban vs rural). These included descriptions of their gender, age group, educational level, marital status and religion. We also included an assessment of their household wealth based on ownership of assets. This variable was constructed from R6 survey data, with methods similar to those used in Demographic and Health Surveys.^40^ It allowed classifying household in wealth quintiles. We also reported the proportion of caregivers who had ever heard messages (from any source) about the need to register births. We tested for differences in the distribution of these characteristics between urban and rural areas using χ^2^ tests.

Our analyses of DCE data relied on the assumption that caregivers are rational actors, who make choices that maximise their individual utility.^41^ The utility *U* that a DCE respondent *r* derives from selecting alternative *i* in a choice set *t* was specified as:

(1) $$U_{r,i,t}=\beta_{r}X_{i,t}+\varepsilon_{r,i,t}$$

where $X_{i,t}$ is a vector of variables describing the attributes of an alternative; *β*_*r*_ is a vector of coefficients that represent the marginal utility that respondents derive from each level of these attributes (their ‘preferences’); and *ε*_*r, i, t*_ is an unobserved error term that is assumed to be independent of individual preferences and attribute levels.^42^ Given a respondent’s preferences, the probability of selecting alternative *i* among a set of *J* alternatives in a choice set is described by a logit model^42 43^:

(2) $$Pr\left(select\;alternative\;i\;in\;choice\;set\;t|\beta_{r}\right)=\frac{e^{\beta_{r}X_{i,t}}}{\sum_{j=1}^{J}e^{\beta_{r}X_{j,t}}}$$

DCE data have often been analysed using conditional logit models,^44 45^ which assume that (A) there is no heterogeneity in preferences across respondents, and (B) there is no correlation among the multiple choices made by the same individual. In this paper, we relaxed these strong assumptions. We used mixed logit models, in which the parameter estimates can be written as the sum of their population average, *b*, and a term $\eta_{r}$ that represents individual deviations from this average,^42^ so that:

(3) $$U_{r,i,t}=\left(b+\eta_{r}\right)X_{i,t}+\varepsilon_{r,i,t}$$

In our models, the $X_{i,t}$ vector included all the attributes listed in table 1. We treated costs, distances and waiting times as continuous variables, expressed in birr, walking time and hours, respectively. Other attributes were treated as categorical variables and were dummy coded,^43^ that is, with a reference category taking value 0. We also included an opt-out constant, that is, a dummy variable taking value 1 if the alternative was not to select any of the two randomly selected facilities included in each choice set,^34 35^ and 0 otherwise. We used the mixlogit command in Stata^46^ to estimate mean coefficients (*b*) and their SDs, along with 95% CIs. We also tested the null hypothesis that all SDs were jointly equal to 0 (likelihood ratio test).

Despite the stratified sampling scheme of the R6 survey, we report unweighted analyses of DCE data. We do so because our analyses were stratified by urban versus rural place of residence, which were the main domains for which estimates were sought in the R6 survey. In addition, the survey weights are not related to the dependent variable in our mixed logit models (ie, DCE choices). Unweighted estimates are thus unbiased and more efficient than weighted estimates.^47^

To further understand respondents’ preferences for various attributes of registration facilities, we conducted a willingness-to-pay (WTP) analysis. We divided the coefficient of each variable obtained using mixed logit models by minus one times the coefficient associated with registration costs.^48^ This allowed standardising the relative utility derived from registering a birth at a facility with a given level of an attribute against costs. All WTP estimates were computed in birr (ie, the local currency in Ethiopia), and translated into US$ using the exchange rate on 1 January 2019.

## Results

We selected 840 caregivers for the birth registration study and 715 consent to participate (response rate=85.1%). Among those, 705 completed the DCE section of the interview. Four hundred and fifty-nine DCE respondents resided in urban areas (65.1%, table 2) versus 246 in rural areas (34.9%).

**Table 2:** Characteristics of participants in the discrete choice experiment, Addis Ababa and SNNPR of Ethiopia 2018/2019

Urban areas		Rural areas		P value*
n	%†	n	%†	
Region				–
Addis Ababa	194	42.3	–	–
SNNPR	265	57.7	246	100.0
Gender				0.024
Men	17	3.7	2	0.8
Women	442	96.3	244	99.2
Age				<0.001
<20	9	2.0	12	4.9
20–29	230	50.1	89	36.2
30–39	183	39.9	100	40.7
40–49	30	6.5	31	12.6
≥50	7	1.5	14	5.7
Marital status				0.172
Never married	13	2.9	3	1.3
Currently married	389	85.7	215	90.3
Previously married	52	11.4	20	8.4
Education				<0.001
No school	50	10.9	122	49.6
Primary level	154	33.5	108	43.9
Secondary level	128	27.9	9	3.7
Higher education	67	14.6	2	0.8
Technical training	60	13.1	5	2.0
Household wealth				<0.001
Poorest quintile	2	0.4	66	26.8
Poorer quintile	4	0.9	71	28.9
Middle quintile	13	2.8	65	26.4
Richer quintile	131	28.5	42	17.1
Richest quintile	309	67.3	2	0.8
Religion				<0.001
Orthodox	220	47.9	34	13.8
Protestant	158	34.4	177	72.0
Muslim	77	16.8	31	12.6
Other	4	0.9	4	1.6
Ever heard messages about birth registration				<0.001
No	209	45.5	188	76.4
Yes	229	49.9	41	16.7
Don’t know	21	4.6	17	6.9

*P values are derived from a χ^2^ test of the association between place of residence and each respondent characteristic.

†Figures in the table are column percentages.

SNNPR, Southern Nations, Nationalities, and People’s Region.

In urban areas, approximately 4 out of 10 respondents resided in Addis Ababa (194/459, 42.3%). All rural respondents resided in the SNNPR. There were large differences in background characteristics by place of residence. Urban respondents were younger, more educated and often members of wealthier households than rural residents. Only 10.9% of urban respondents (50/459) had never been to school versus 49.6% of rural respondents (122/246). Similarly, more than two-thirds of urban respondents resided in a household that belonged to the wealthiest quintile of the Ethiopian population (314/465, 67.5%), whereas this was the case for <1% of rural respondents (2/250, 0.8%). A larger proportion of urban respondents had ever heard messages about birth registration (49.9% vs 16.7%). DCE participants were predominantly women (96.3% in urban areas and 99.2% in rural areas), who were currently married (85.7% in urban areas and 90.3% in rural areas).

Participants failed to complete 26 of the 5640 total DCE choices they were asked to make (0.45%). Among valid DCE answers, respondents opted out of the choice between the two hypothetical facilities 726 out of 5614 times (12.9%).

This proportion was slightly higher in rural areas (295/1960, 15.1%) than in urban areas (431/3654, 11.8%). Two-thirds of respondents never opted out of the choice they were asked to make (470/705, 66.7%). This proportion was higher among urban respondents than among rural respondents (69.5% vs 61.4%). Only six respondents (0.85%) opted out of every choice. Among those, five resided in urban areas and one resided in rural areas. In the choice set with a dominant option, 656 respondents selected the objectively most desirable registration facility (out of 703 respondents having provided valid answers to this choice set, 93.3%). There were no differences in the likelihood of selecting the dominant option between urban and rural respondents (93.2% in urban areas vs 93.5% in rural areas).

The parameter estimates for the effects of registration facility attributes on utility are shown in table 3. Positive coefficient estimates indicate that respondents favour a particular attribute or level of that attribute. Conversely, negative estimates indicate that an attribute and/or its level create disutility for the respondents.

**Table 3:** Results from random parameter logit models of DCE data, Addis Ababa and SNNPR of Ethiopia 2018/2019

	Urban areas		Rural areas	
	Estimate	95% CI	Estimate	95% CI
**Coefficients (*****b*****)**				
Opt-out constant	−6.310***	−7.004 to −5.616	−6.769***	−7.682 to −5.858
Cost of certificate (birr)	−0.010***	−0.011 to −0.009	−0.020***	−0.023 to −0.017
Waiting time (hours)	−0.130***	−0.174 to −0.086	−0.127***	−0.190 to −0.063
Distance (hours)	−0.581***	−0.664 to −0.499	−0.569***	−0.669 to −0.470
Number of visits				
Multiple visits	Ref	–	Ref	–
Single visit	0.731***	0.595 to 0.866	0.631***	0.459 to 0.803
Opening hours				
Regular hours	Ref	–	Ref	–
Extended hours	0.205**	0.054 to 0.355	0.132	−0.071 to 0.335
Weekend hours	0.555***	0.384 to 0.725	0.543***	0.298 to 0.787
Applicants				
Both parents	Ref	–	Ref	–
Only one parent	0.427***	0.266 to 0.588	0.174	−0.020 to 0.368
**SDs**				
Opt-out constant	3.290***	2.722 to 3.858	3.316***	2.487 to 4.145
Cost of certificate (birr)	0.007***	0.006 to 0.009	0.012***	0.010 to 0.015
Waiting time (hours)	0.224***	0.154 to 0.294	0.217***	0.125 to 0.311
Distance (hours)	0.590***	0.498 to 0.683	0.449***	0.334 to 0.564
Number of visits				
Multiple visits	Ref	–	Ref	–
Single visit	0.527***	0.305 to 0.748	0.061	−0.026 to 0.382
Opening hours				
Regular hours	Ref	–	Ref	–
Extended hours	0.063	−0.367 to 0.492	0.337	−0.191 to 0.864
Weekend hours	0.092	−0.551 to 0.736	0.023	−0.439 to 0.486
Applicants				
Both parents	Ref	–	Ref	–
Only one parent	0.931***	0.695 to 1.166	0.121	−0.381 to 0.624
**Model diagnostics**	**Both parents**		**Only one parent**	
Respondents, n	459		246	
Choice sets, n	3654		1960	
Log likelihood	−2516.218		−1238.431	
Likelihood ratio χ^2^	1039.12***		651.83***	

Mixed logit models were fitted using the mixlogit command in Stata, from 500 Halton draws. SD refers to the standard deviation of the parameter estimate.

The likelihood ratio χ^2^ tests the null hypothesis that all SDs are jointly equal to zero. This null hypothesis is rejected for both the urban and rural areas.

***P<0.001; **p<0.01; *p<0.05.

DCE, discrete choice experiment; SNNPR, Southern Nations, Nationalities, and People’s Region.

In both urban and rural areas, respondents were less likely to select facilities that had longer waiting times (*β*=−0.130 in urban areas, and *β*=−0.127 in rural areas), or were located further away from their residence (*β*=−0.581 in urban areas, and *β*=−0.569 in rural areas). They preferred facilities that completed all registration procedures and delivered birth certificates in a single visit (*β*=0.731 in urban areas, and *β*=0.631 in rural areas) and facilities that were open on weekends (*β*=0.555 in urban areas, and *β*=0.543 in rural areas).

Higher costs of birth certificates negatively affected the utility of caregivers in both urban and rural areas (*β*=−0.010 and *β*=−0.020, respectively). However, the disutility resulting from higher costs was larger in rural areas. Respondents in urban areas expressed preferences for facilities that were open for extended hours on weekdays (*β*=0.205), and that only required one of the parents to be present for registration (*β*=0.427). Respondents in rural areas did not display similar preferences in their choices.
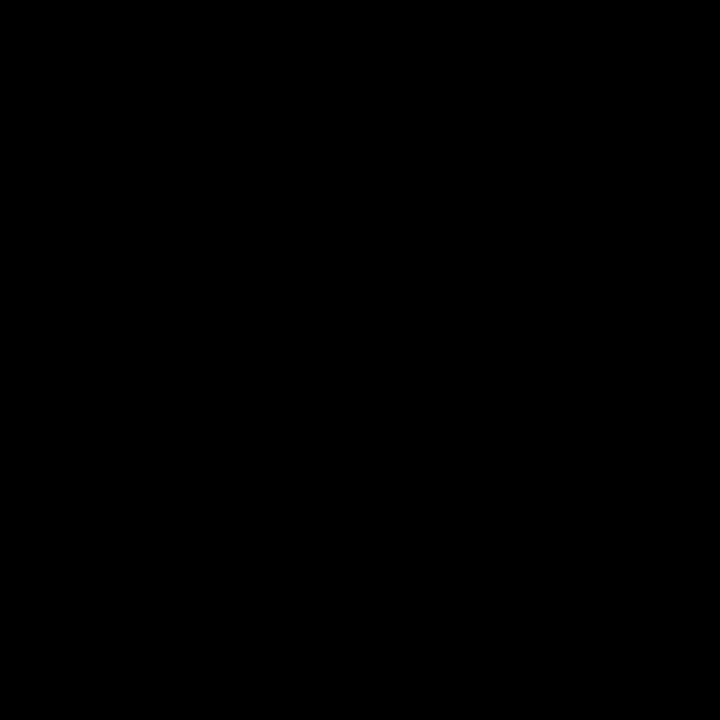


There was heterogeneity between respondents in preferences relating to several attributes (table 3). We rejected the null hypothesis that there was no variation across participants in the effects of costs (SD=0.007 in urban areas, and SD=0.012 in rural areas), waiting time (SD=0.224 in urban areas, and SD=0.217 in rural areas) and distance (SD=0.590 in urban areas, and SD=0.449 in rural areas) on choice patterns. In urban areas, there was heterogeneity in preferences related to the number of visits required to register a birth (SD=0.527), and with application procedures (SD=0.931). There was no evidence of heterogeneity in preferences relating to the opening hours of the registration facility, both in urban and rural areas. In rural areas, there was no evidence of heterogeneity in preferences relating to the number of visits required to obtain the birth certificate and to application procedures.

Results from the WTP analysis are shown in table 4. Negative WTP estimates indicate that respondents would require compensation to select registration facilities with such attribute levels, whereas positive WTP estimates represent the implicit price that respondents are willing to incur to access registration facilities with an attractive attribute. DCE participants would thus require compensation to use registration facilities that are further away from their residence (WTP=−57.79 birr or −US$2.02 per additional hour in urban areas, and WTP=−28.37 birr or −US$1.00 in rural areas), or that require longer waiting times (WTP=−12.93 birr or −US$0.45 per additional hour in urban areas, and WTP=−6.31 birr or −US$0.22 in rural areas). Participants would be willing to incur a cost of 72.61 birr (US$2.54) in urban areas, and 31.44 birr (US$1.10) in rural areas, to access a facility that delivered the birth certificate in a single visit. Relative to a facility open at regular weekday hours, participants were willing to incur additional costs in order to attend a registration facility that opens on weekends (55.15 birr or US$1.93 in urban areas, vs 27.02 birr or US$0.95 in rural areas).

**Table 4:** Estimates of willingness to pay for facility attributes from mixed logit models, Addis Ababa and SNNPR of Ethiopia 2018/2019

Attribute	Urban areas		Rural areas	
	WTP*	95% CI	WTP*	95% CI
Waiting time (hours)	−12.93	−17.35 to −8.51	−6.31	−9.55 to −3.06
Distance (hours)	−57.79	−66.47 to −49.11	−28.37	−33.05 to −23.69
Number of visits				
Multiple visits	Ref	–	Ref	–
Single visit	72.61	58.05 to 87.17	31.44	22.05 to 40.82
Opening hours				
Regular hours	Ref	–	Ref	–
Extended hours	20.33	5.54 to 35.13	2.69	−8.52 to 13.90
Weekend hours	55.15	38.60 to 71.70	27.02	14.51 to 39.54
Applicants				
Both parents	Ref	–	Ref	–
Only one parent	42.43	25.47 to 59.39	8.66	−1.12 to 18.45

*The coefficients are expressed in birr (1 birr=US$0.035 as of 1 January 2019). A positive coefficient represents the amount that respondents are willing to contribute to access a facility with a given level of an attribute. Negative coefficients indicate that respondents should be compensated in order to offset the disutility due to a specific level of an attribute.

SNNPR, Southern Nations, Nationalities, and People’s Region; WTP, willingness to pay.

## Discussion

We documented the preferences of caregivers in registering births in two regions of Ethiopia. We used an established experimental survey method (ie, a DCE) in a population-based sample. The DCE confirmed the existence of significant barriers to birth registration in these two regions. DCE participants were less likely to opt for registration facilities that were further away from their homes, that had longer wait times to obtain services and/or that charged a fee for the acquisition of the birth certificate. This is consistent with findings from other studies that have investigated barriers to birth registration in other settings, using an array of other methodologies.^49 50^

We found strong preferences for registration facilities that deliver a birth certificate in a single registration visit. The current CRVS policy in Ethiopia requires that the administrative facilities implementing birth registration deliver the birth certificate immediately to the parents/caregivers. However, in our survey, more than a third of caregivers who had registered the birth of their child reported having to return to the registration facility several times to complete that process. More consistently implementing the current policy might help improve registration rates.

DCE respondents in urban areas also preferred registration facilities that only required one of the two parents to be present at the time of registration. The main procedure outlined by the current legal framework in Ethiopia however requires both parents to be present at the registration facility in order to register a birth. This might constitute a barrier to birth registration, as also indicated by prior studies that have investigated reasons reported by caregivers for not registering a birth in other settings.^50^ Indeed, this might make birth registration more complex for children who have at least one parent with rigid work schedules or who is engaged in migration, or for children whose parents might no longer be in a relationship/union.

Our study highlighted other characteristics of registration facilities that might play a key role in the registration-related behaviours of caregivers. In particular, choices were influenced by the opening schedule of the facility: DCE participants expressed consistent preferences for registration facilities that remained opened on weekends. In urban areas, they also expressed preferences for facilities that remained opened for extended hours on weekdays. This might be because the current opening schedule of registration facilities conflicts with work schedules or with times during which economic activities of caregivers are ongoing.

There were differences in the preferences revealed by the DCE between residents of urban and rural areas. In particular, the effects of costs on patterns of choices were larger in rural areas than in urban areas. This is likely due to the fact that rural residents were much poorer than urban residents. As a result, WTP estimates were lower in rural areas. For example, caregivers in urban areas were willing to pay more than 55 birr (ie, approximately US$2.0 on 1 January 2019) to access a registration facility that was opened on weekends, whereas caregivers in rural areas were willing to incur only half of that implicit price (27.02 birr or US$0.99).

Our DCE has several limitations. First, we only investigated the main effects of each attribute of registration facilities, without considering potential interactions between attributes. This is problematic because the effects of an attribute might depend on the levels of another: for example, facilities that remain open on weekends might be particularly attractive in settings where both parents are required to be present at the time of registration, because it is more likely that both parents will be available on weekends. Investigating interactions between attributes would however require respondents to make a larger number of choices during the DCE than we deemed feasible in this setting.^51^

Second, due to limited sample sizes, we only investigated whether preferences for birth registration varied between urban and rural areas. We did not investigate whether preferences varied across other subgroups, for example, by poverty or educational level. In urban areas, we also did not investigate whether preferences varied between Addis Ababa and the smaller cities of the SNNPR. Third, we only presented DCE respondents with choices that were characterised by a limited set of attributes. Other aspects of the registration facilities/process might affect registration choices, for example, whether the registration office is located in an administrative setting or in a healthcare setting.

Fourth, some of the choice patterns in our DCE indicated that some respondents might not have fully understood the choices they were asked to make, or experienced fatigue. A small fraction of the respondents consistently opted out of the choices they were presented (<1%); whereas others (6.7%) failed to select the objectively most appealing alternative in a dominant choice set. However, these proportions were consistent with the experience of other high-quality DCEs,^36^ including with more educated populations.^20 52^ We also replicated our analyses of the DCE after excluding respondents with inconsistent choices, and we found similar patterns of preferences (online supplementary file 2).

10.1136/bmjgh-2019-002209.supp2 Supplementary data

Fifth, the caregivers we interviewed were in large majority women (>96%). However, as for other services (eg, family planning), men likely play important roles in the decision process about birth registration, in particular in settings where their presence is required for registration. Future studies should thus ensure that men are included in DCEs designed to elicit preferences towards birth registration. Sixth, our statistical analyses of DCE data made several assumptions that might have impacted our results. For example, we used mixed logit models to represent heterogeneity in preferences within the population. Other recent work has used latent class models to represent such heterogeneity.^23 43^ Similarly, we accounted for opt-out effects by including an alternative specific constant in our models. Other approaches (eg, nested logit models) might yield slightly different estimates of the WTP for various attributes of a service.^34^

Finally, our work was limited to two regions of Ethiopia, and thus does not represent the preferences of residents of other regions of the country where birth registration is also low. Furthermore, Ethiopia is a country where birth registration has only recently been reorganised and implemented nationwide. Preferences for birth registration might differ in countries with higher background rates of event registration (eg, Kenya).

Despite these limitations, our work indicates several strategies that might help further accelerate the scale-up of birth registration in Addis Ababa and in the SNNPR. It appears warranted to explore whether altering the opening schedule of registration facilities to allow evening and weekend openings might help improve birth registration rates. This is feasible within the current legislative framework for birth registration in Ethiopia and could thus be tested during a cluster-randomised trial in those two regions. Such a change might stimulate the demand for birth registration and complement initiatives that aim to strengthen and streamline the administrative systems that implement civil registration.^14^ Other strategies highlighted by our DCE (eg, reducing the legal requirements for parental presence at the time of registration) might also have an impact on birth registration rates, but would not be possible without amendments to the legislative framework that regulates birth registration in Ethiopia. Finally, our work shows that DCEs might also be a useful methodology to help orient initiatives to increase birth registration rates, similar to their role in health systems strengthening.

## References

[1] Setel PW, Macfarlane SB, Szreter S, et al A scandal of invisibility: making everyone count by counting everyone. Lancet 2007;370:1569–77. 10.1016/S0140-6736(07)61307-5 17992727

[2] Cappa C, Gregson K, Wardlaw T, et al Birth registration: a child's passport to protection. Lancet Glob Health 2014;2:e67–8. 10.1016/S2214-109X(13)70180-3 25104657

[3] AbouZahr C, de Savigny D, Mikkelsen L, et al Towards universal civil registration and vital statistics systems: the time is now. Lancet 2015;386:1407–18. 10.1016/S0140-6736(15)60170-2 25971217

[4] Fagernäs S, Odame J Birth registration and access to health care: an assessment of Ghana's campaign success. Bull World Health Organ 2013;91:459–64. 10.2471/BLT.12.111351 24052683 PMC3777139

[5] Brito S, Corbacho A, Osorio R Does birth under-registration reduce childhood immunization? Evidence from the Dominican Republic. Health Econ Rev 2017;7:14. 10.1186/s13561-017-0149-3 28337738 PMC5364131

[6] Mikkelsen L, Phillips DE, AbouZahr C, et al A global assessment of civil registration and vital statistics systems: monitoring data quality and progress. Lancet 2015;386:1395–406. 10.1016/S0140-6736(15)60171-4 25971218

[7] Phillips DE, Adair T, Lopez AD How useful are registered birth statistics for health and social policy? a global systematic assessment of the availability and quality of birth registration data. Popul Health Metr 2018;16:21. 10.1186/s12963-018-0180-6 30587201 PMC6307230

[8] Nomura M, Xangsayarath P, Takahashi K, et al Socioeconomic determinants of accessibility to birth registration in Lao PDR. BMC Public Health 2018;18:116. 10.1186/s12889-017-5009-x 29310660 PMC5759213

[9] Amo-Adjei J, Annim SK Socioeconomic determinants of birth registration in Ghana. BMC Int Health Hum Rights 2015;15:14. 10.1186/s12914-015-0053-z 26072313 PMC4465725

[10] Bhatia A, Ferreira LZ, Barros AJD, et al Who and where are the uncounted children? inequalities in birth certificate coverage among children under five years in 94 countries using nationally representative household surveys. Int J Equity Health 2017;16:148. 10.1186/s12939-017-0635-6 28821291 PMC5562988

[11] Mills S, Lee JK, Rassekh BM Benefits of linking civil registration and vital statistics with identity management systems for measuring and achieving sustainable development goal 3 indicators. J Health Popul Nutr 2019;38:18. 10.1186/s41043-019-0178-0 31627734 PMC6800484

[12] Sankoh O, Dickson KE, Faniran S, et al Births and deaths must be registered in Africa. Lancet Glob Health 2020;8:e33–4. 10.1016/S2214-109X(19)30442-5 31839137

[13] de Savigny D, Riley I, Chandramohan D, et al Integrating community-based verbal autopsy into civil registration and vital statistics (CRVS): system-level considerations. Glob Health Action 2017;10:1272882. 10.1080/16549716.2017.1272882 28137194 PMC5328373

[14] Cobos Muñoz D, Abouzahr C, de Savigny D The 'Ten CRVS Milestones' framework for understanding Civil Registration and Vital Statistics systems. BMJ Glob Health 2018;3:e000673. 10.1136/bmjgh-2017-000673 PMC5873547 29607102

[15] Central Statistical Agency - CSA/Ethiopia, ICF Ethiopia Demographic and Health Survey 2016 [Internet]. Addis Ababa, Ethiopia: CSA and ICF, 2017 http://dhsprogram.com/pubs/pdf/FR328/FR328.pdf

[16] Mangham LJ, Hanson K, McPake B How to do (or not to do) … Designing a discrete choice experiment for application in a low-income country. Health Policy Plan 2009;24:151–8. 10.1093/heapol/czn047 19112071

[17] Zwerina K Introduction. in: Zwerina K, editor. discrete choice experiments in marketing: use of Priors in efficient choice designs and their application to individual preference measurement. Heidelberg: Physica-Verlag HD, 1997.

[18] Lagarde M, Blaauw D A review of the application and contribution of discrete choice experiments to inform human resources policy interventions. Hum Resour Health 2009;7:62. 10.1186/1478-4491-7-62 19630965 PMC2724490

[19] Kruk ME, Paczkowski M, Mbaruku G, et al Women's preferences for place of delivery in rural Tanzania: a population-based discrete choice experiment. Am J Public Health 2009;99:1666–72. 10.2105/AJPH.2008.146209 19608959 PMC2724466

[20] Kruk ME, Johnson JC, Gyakobo M, et al Rural practice preferences among medical students in Ghana: a discrete choice experiment. Bull World Health Organ 2010;88:333–41. 10.2471/BLT.09.072892 20458371 PMC2865662

[21] Kruk ME, Riley PL, Palma AM, et al How can the health system retain women in HIV treatment for a lifetime? a discrete choice experiment in Ethiopia and Mozambique. PLoS One 2016;11:e0160764. 10.1371/journal.pone.0160764 27551785 PMC4994936

[22] Larson E, Vail D, Mbaruku GM, et al Moving toward patient-centered care in Africa: a discrete choice experiment of preferences for delivery care among 3,003 Tanzanian women. PLoS One 2015;10:e0135621. 10.1371/journal.pone.0135621 26262840 PMC4532509

[23] Abdel-All M, Angell B, Jan S, et al What do community health workers want? findings of a discrete choice experiment among accredited social health activists (ASHAs) in India. BMJ Glob Health 2019;4:e001509. 10.1136/bmjgh-2019-001509 PMC6570975 31263591

[24] Zimmerman L, Olson H, et al PMA2020: rapid Turn-Around survey data to monitor family planning service and practice in ten countries. Stud Fam Plann 2017;48:293–303. 10.1111/sifp.12031 28885679 PMC6084342

[25] Uganda Bureau of Statistics (UBOS) & ICF. Uganda Demographic and Health Survey, 2016 Available: https://dhsprogram.com/publications/publication-fr333-dhs-final-reports.cfm

[26] Kenya national Bureau of statistics. Kenya demographic and health survey, 2014 Available: https://dhsprogram.com/publications/publication-fr308-dhs-final-reports.cfm

[27] Shiferaw S, Spigt M, Seme A, et al Does proximity of women to facilities with better choice of contraceptives affect their contraceptive utilization in rural Ethiopia? PLoS One 2017;12:e0187311. 10.1371/journal.pone.0187311 29131860 PMC5683563

[28] Tadele A, Abebaw D, Ali R Predictors of unmet need for family planning among all women of reproductive age in Ethiopia. Contracept Reprod Med 2019;4:6. 10.1186/s40834-019-0087-z 31171978 PMC6547515

[29] Vital events registration kicks off in Ethiopia. UNICEF Ethiopia, 2016 Available: https://unicefethiopia.org/2016/08/04/vital-events-registration-kicks-off-in-ethiopia/

[30] Centre of Excellence for CRVS systems Snapshot of civil registration and vital statistics systems of Ethiopia, 2018 Available: https://crvssystems.ca/sites/default/files/assets/files/CRVS_EthiopiaSnapshot_e.pdf

[31] Assessment of the status of birth registration in Gamo Gofa zone and Konso Woreda, SNNPR, Ethiopia, 2019 Available: https://www.researchsquare.com/article/rs-3051/v1

[32] Hole A DCREATE: Stata module to create efficient designs for discrete choice experiments. 2017. (statistical software components). Available: https://econpapers.repec.org/software/bocbocode/s458059.htm

[33] de Bekker-Grob EW, Hol L, Donkers B, et al Labeled versus unlabeled discrete choice experiments in health economics: an application to colorectal cancer screening. Value Health 2010;13:315–23. 10.1111/j.1524-4733.2009.00670.x 19912597

[34] Campbell D, Erdem S Including Opt-Out options in discrete choice experiments: issues to consider. Patient 2019;12:1–14. 10.1007/s40271-018-0324-6 30073482

[35] Veldwijk J, Lambooij MS, de Bekker-Grob EW, et al The effect of including an opt-out option in discrete choice experiments. PLoS One 2014;9:e111805. 10.1371/journal.pone.0111805 25365169 PMC4218820

[36] Tervonen T, Schmidt-Ott T, Marsh K, et al Assessing rationality in discrete choice experiments in health: an investigation into the use of dominance tests. Value Health 2018;21:1192–7. 10.1016/j.jval.2018.04.1822 30314620

[37] Kruk ME, Paczkowski MM, Tegegn A, et al Women's preferences for obstetric care in rural Ethiopia: a population-based discrete choice experiment in a region with low rates of facility delivery. J Epidemiol Community Health 2010;64:984–8. 10.1136/jech.2009.087973 19822558

[38] de Bekker-Grob EW, Donkers B, Jonker MF, et al Sample size requirements for Discrete-Choice experiments in healthcare: a practical guide. Patient 2015;8:373–84. 10.1007/s40271-015-0118-z 25726010 PMC4575371

[39] Hartung C, Lerer A, Anokwa Y, et al Open data kit: tools to build information services for developing regions. in: proceedings of the 4th ACM/IEEE International Conference on information and communication technologies and development – ICTD. P. 1–12. Available: http://dl.acm.org/citation.cfm?doid=2369220.2369236

[40] Vyas S, Kumaranayake L Constructing socio-economic status indices: how to use principal components analysis. Health Policy Plan 2006;21:459–68. 10.1093/heapol/czl029 17030551

[41] McFadden D The choice theory approach to market research. Marketing Science 1986;5:275–97. 10.1287/mksc.5.4.275

[42] Revelt D, Train K Mixed Logit with repeated choices: households' choices of appliance efficiency level. Rev Econ Stat 1998;80:647–57. 10.1162/003465398557735

[43] Hauber AB, González JM, Groothuis-Oudshoorn CGM, et al Statistical methods for the analysis of discrete choice experiments: a report of the ISPOR conjoint analysis good research practices Task force. Value Health 2016;19:300–15. 10.1016/j.jval.2016.04.004 27325321

[44] McFadden DL Chapter 24 Econometric analysis of qualitative response models. in: Handbook of Econometrics. Elsevier 1984:1395–457.

[45] Maddala GS Limited-dependent and qualitative variables in econometrics. Cambridge university press, 1986.

[46] Hole AR Fitting mixed Logit models by using maximum simulated likelihood. Stata J 2007;7:388–401. 10.1177/1536867X0700700306

[47] Winship C, Radbill L Sampling Weights and Regression Analysis: Sociological Methods & Research 1994;23:230–57.

[48] Hole A WTP: Stata module to estimate confidence intervals for willingness to pay measures, 2007 Available: https://econpapers.repec.org/software/bocbocode/s456808.htm 10.1002/hec.1197 17238222

[49] Duff P, Kusumaningrum S, Stark L Barriers to birth registration in Indonesia. Lancet Glob Health 2016;4:e234–5. 10.1016/S2214-109X(15)00321-6 27013307

[50] Fisker AB, Rodrigues A, Helleringer S Differences in barriers to birth and death registration in Guinea-Bissau: implications for monitoring national and global health objectives. Trop Med Int Health 2019;24:166–74. 10.1111/tmi.13177 30430696

[51] Reed Johnson F, Lancsar E, Marshall D, et al Constructing experimental designs for discrete-choice experiments: report of the ISPOR conjoint analysis experimental design good research practices Task force. Value Health 2013;16:3–13. 10.1016/j.jval.2012.08.2223 23337210

[52] Rockers PC, Jaskiewicz W, Kruk ME, et al Differences in preferences for rural job postings between nursing students and practicing nurses: evidence from a discrete choice experiment in Lao people's Democratic Republic. Hum Resour Health 2013;11:22. 10.1186/1478-4491-11-22 23705805 PMC3671159

