# Supplementary material for: Improving the demand for birth registration: a discrete choice experiment in Ethiopia
Source: BMJ Glob Health. 2020 May 21;5(5):e002209. doi: 10.1136/bmjgh-2019-002209 (PMC7247413; doi:10.1136/bmjgh-2019-002209)
Supplement: Supplementary data [file bmjgh-2019-002209supp001.pdf]

Systematic literature review of barriers to birth registration in low- and middle-income countries

Dana Sarnak, Stephane Helleringer, Lori Rosman

Citation

Dana Sarnak, Stephane Helleringer, Lori Rosman. Systematic literature review of barriers to birth registration in low- and middle-income countries. PROSPERO 2018 CRD42018089552 Available from: https://www.crd.york.ac.uk/prospero/display_record.php?ID=CRD42018089552

Review question

The overall aim of this review is to synthesize the existing literature to understand levels and barriers to birth registration in low- and middle-income countries.

Searches

We will search PubMed (1946-present), Embase (1947-present), Scopus (1788-present), Africa-Wide Information (1700s-present), Global Health (1910-preseent), PAIS International (1977-present), Popline (1970-present), Latin American and Caribbean Health Sciences Literature Database (LILACS) (1982-present), and the World Health Organization Global Health Library (inception-present).

We will use a combination of controlled vocabulary and keyword terms customized for each database to search the concepts of “birth registration” and “low and middle income countries.” We will not use any date or language restrictions in our searches. Searches will be developed by an experienced informationist with input from the research team.

To find additional articles, the authors will hand search the reference list of the key articles located through the database search.

We will also search the internet for technical reports from government agencies, key NGOs or scientific research groups, working papers from research groups or committees, white papers, preprints, conference proceedings and dissertations.

Types of study to be included

Included:
-Peer review journal research articles- quantitative and qualitative studies that aim to discover barriers and facilitators to birth registration

-Technical reports from government agencies, key NGOs or scientific research groups

-Working papers from research groups or committees

-White papers

-Conference proceedings

-Dissertations

Condition or domain being studied

We are studying the determinants of birth registration. Birth registration is “the process by which a child’s birth is recorded in the civil register by the government authority” and provides legal recognition, birth certificate, and as a result many other legal documents and rights. (UNICEF).

Improving birth registration is a target under SDGs (Target 16.9: By 2030, provide legal identity for all, including birth registration). Yet, birth registration rates in low- and middle- income countries remain low, for example In Ethiopia, data from the 2016 Demographic and Health Surveys showed only 3% of children

under age five years of age had their birth registered at the national level.

Participants/population

The review focuses on low and middle-income countries as classified by the World Bank LMIC country classification.

Intervention(s), exposure(s)

All variations of barriers to birth registration encountered by individuals, communities and countries.

Comparator(s)/control

Considering that there may be contextual differences in across countries, comparison groups (if any) may vary depending on determinants/challenges to birth registration access.

Context

Main outcome(s)

Outcomes regarding the following will be considered:

-Supply side barriers to birth registration. For example, supply side barriers include health system barriers that include geographic location of birth registration, costs of birth registration and administrative barriers to birth registration.

-Demand side barriers to birth registration. For example, demand side barriers include individual- or interpersonal-level barriers such as knowledge and attitudes towards birth registration.

These outcomes will be measured in the study through quantitative data collection such as surveys of individuals, or in qualitative data collection including interviews with key stakeholders.

Additional outcome(s)

None

Data extraction (selection and coding)

Following screening of article titles and abstracts for eligibility, data extraction will be guided by a data abstraction form. Information, such as author details, journal, year of publication, location of study, sample size, response rate and population characteristics, will be noted. Factors reported as barriers/challenges to birth registration will be collected and pooled.

Risk of bias (quality) assessment

The Preferred Reporting Items for Systematic Reviews and Meta-Analyses (PRISMA) checklist, by Moher et al. (2009), will guide the review process. Two independent investigators will review the full set of titles and abstracts to decide which articles will be included based on predetermined criteria. Two investigators will independently review all full-text articles deemed eligible and consensus reached on any differences arising through discussions.

Because we are not evaluating clinical trials, we will need to adapt our risk of bias tools from established methodologies. We plan to assess quantitative methods of articles by evaluating traditional domains such as the selection of participants, confounding variables, measurement, incomplete outcome data, and selective outcome reporting.

Qualitative research will also be evaluated by adaptions of established tools to evaluate quality assessment, for example using the Critical Appraisal Skills Programme (CASP) tool.

Strategy for data synthesis

Given the nature and likely heterogeneity of the outcomes, summary of data will be carried out using

narrative, qualitative synthesis of the barriers to birth registration.

We will organize outcomes using an adapted supply-demand analytical framework from Jacobs et al 2012*.

*Jacobs B, Ir P, Bigdeli M, Annear PL, Van Damme W. Addressing access barriers to health services: an analytical framework for selecting appropriate interventions in low-income Asian countries. Health Policy Plan. 2012 Jul;27(4):288-300. doi: 10.1093/heapol/czr038. Epub 2011 May 12. Review. PubMed PMID: 21565939.

Analysis of subgroups or subsets
None planned

Contact details for further information
Dr. Stephane Helleringer
sheller7@jhu.edu

Organisational affiliation of the review
Johns Hopkins Bloomberg School of Public Health
Dr. Stephane Helleringer

Review team members and their organisational affiliations
Miss Dana Sarnak. Johns Hopkins Bloomberg School of Public Health
Professor Stephane Helleringer. Johns Hopkins Bloomberg School of Public Health
Ms Lori Rosman. Welch Medical Library

Type and method of review
Systematic review

Anticipated or actual start date
15 February 2018

Anticipated completion date
15 September 2018

Funding sources/sponsors
The Bill and Melinda Gates Foundation

Conflicts of interest

Language
English

Country
United States of America

Stage of review
Review Ongoing

Subject index terms status
Subject indexing assigned by CRD

Subject index terms
Female; Humans; Income; Parturition; Pregnancy; Publications

Date of registration in PROSPERO
18 April 2018

Date of publication of this version
18 April 2018

Details of any existing review of the same topic by the same authors

None

Stage of review at time of this submission

Stage	Started	Completed
Preliminary searches	Yes	No
Piloting of the study selection process	Yes	No
Formal screening of search results against eligibility criteria	No	No
Data extraction	No	No
Risk of bias (quality) assessment	No	No
Data analysis	No	No

Versions

18 April 2018

PROSPERO
This information has been provided by the named contact for this review. CRD has accepted this information in good faith and registered the review in PROSPERO. The registrant confirms that the information supplied for this submission is accurate and complete. CRD bears no responsibility or liability for the content of this registration record, any associated files or external websites.
