# Supplementary material for: Improving the demand for birth registration: a discrete choice experiment in Ethiopia
Source: BMJ Glob Health. 2020 May 21;5(5):e002209. doi: 10.1136/bmjgh-2019-002209 (PMC7247413; doi:10.1136/bmjgh-2019-002209)
Supplement: Supplementary data [file bmjgh-2019-002209supp002.pdf]

## SUPPLEMENTARY FILE 2: SENSITIVITY ANALYSIS

| | Urban Areas | | Rural Areas | |
|---|---|---|---|---|
| | **Estimate** | **95% CI** | **Estimate** | **95% CI** |
| **Coefficients (b)** | | | | |
| Opt-out constant | -5.828*** | -6.402 to -5.254 | -6.923*** | -7.789 to -6.057 |
| Cost of certificate (in birrs) | -0.009*** | -0.011 to -0.008 | -0.020*** | -0.022 to -0.017 |
| Waiting time (in hours) | -0.108*** | -0.151 to -0.066 | -0.140*** | -0.202 to -0.077 |
| Distance (in hours) | -0.563*** | -0.643 to -0.484 | -0.526*** | -0.618 to -0.433 |
| Number of visits | | | | |
| Multiple visits | Ref | -- | Ref | -- |
| Single visit | 0.739*** | 0.609 to 0.869 | 0.640*** | 0.462 to 0.818 |
| Opening hours | | | | |
| Regular hours | Ref | -- | Ref | -- |
| Extended hours | 0.244** | 0.093 to 0.396 | -0.023 | -0.250 to 0.203 |
| Weekend hours | 0.601*** | 0.426 to 0.776 | 0.495*** | 0.244 to 0.747 |
| Applicants | | | | |
| Both parents | Ref | -- | Ref | -- |
| Only one parent | 0.471*** | 0.328 to 0.613 | 0.154 | -0.047 to 0.355 |
| **Standard deviations (SD)** | | | | |
| Opt-out constant | 2.427*** | 2.024 to 2.830 | 3.022*** | 2.377 to 3.667 |
| Cost of certificate (in birrs) | 0.007*** | 0.006 to 0.008 | 0.012*** | 0.001 to 0.014 |
| Waiting time (in hours) | 0.206*** | 0.135 to 0.276 | 0.166* | 0.025 to 0.308 |
| Distance (in hours) | 0.572*** | 0.483 to 0.661 | 0.417*** | 0.311 to 0.522 |
| Number of visits | | | | |
| Multiple visits | Ref | -- | Ref | -- |
| Single visit | 0.371*** | 0.162 to 0.580 | 0.062 | -0.227 to 0.352 |
| Opening hours | | | | |
| Regular hours | Ref | -- | Ref | -- |
| Extended hours | 0.013 | -0.293 to 0.319 | 0.253 | -0.156 to 0.662 |
| Weekend hours | 0.072 | -0.362 to 0.507 | 0.118 | -0.329 to 0.566 |
| Applicants | | | | |
| Both parents | Ref | -- | Ref | -- |
| Only one parent | 0.463** | 0.128 to 0.798 | 0.233 | -0.165 to 0.631 |
| **Model diagnostics** | | | | |
| Number of choice sets | 3,406 | | 1,832 | |
| Log likelihood | -2,321.473 | | -1,128.773 | |
| Likelihood ratio $\chi_2$ | 844.8*** | | 571.18*** | |

**Table S2-1: Results from mixed logit models of DCE data, including only respondents who correctly selected the preferable facility in the choice set with a dominant option.**
*Notes:* Mixed logit models were fitted using the mixlogit command in Stata, from 500 Halton draws. SD refers to the standard deviation of the parameter estimate. *** p<0.001, **p<0.01, * p<0.05. The likelihood ratio $\chi_2$ tests the null hypothesis that all standard deviations are jointly equal to zero. This null hypothesis is rejected for both the urban and rural areas.
